# Childhood Vitamin D Status and Circulating Insulin-like Peptide 3 as a Marker of Leydig Cell Functional Capacity in Young Adulthood: Findings from the Avon Longitudinal Study of Parents and Children

**DOI:** 10.3390/nu18152426

**Published:** 2026-07-24

**Authors:** Bilal Tulumcu, Richard Ivell, Ravinder Anand-Ivell

**Affiliations:** 1 School of Biosciences, University of Nottingham, Sutton Bonington LE12 5RD, UK; stxbt8@nottingham.ac.uk (B.T.); richard.ivell@nottingham.ac.uk (R.I.); 2 School of Veterinary Medicine and Science, University of Nottingham, Sutton Bonington LE12 5RD, UK

**Keywords:** insulin-like peptide 3 (INSL3), Leydig cells, vitamin D, ALSPAC, male reproductive development, puberty, young adulthood, nutritional epidemiology, testicular function, cohort study

## Abstract

**Background/Objectives:** Circulating insulin-like peptide 3 (INSL3) is a constitutively secreted product of mature Leydig cells and is considered a stable biomarker of Leydig cell functional capacity, a key determinant of androgenic status across the male lifespan. Since the adult Leydig cell population is established during late childhood and puberty, nutritional and metabolic exposures during these periods may contribute to later variation in young adult INSL3. To investigate whether childhood and adolescent nutritional/metabolic markers, particularly vitamin D, fatty acids, amino acids, and dietary trace-element intake, are associated with circulating INSL3 concentrations in young adulthood. **Methods:** Data were analysed from the Avon Longitudinal Study of Parents and Children (ALSPAC), a population-based UK birth cohort. Nutritional and metabolic markers measured during childhood and adolescence included vitamin D, fatty acid-related traits, glutamine, leucine, dietary zinc intake, and dietary selenium intake. Associations with circulating INSL3 at 17 and 24 years were first explored using bivariate correlation analysis. Variables showing signals of interest were then examined in multiple linear regression models using log-transformed data and relevant covariates, including BMI at 8 years and log-transformed exact age at the age-24 clinic visit. **Results:** Among the variables examined, vitamin D at 9 years showed a small positive association with INSL3 at 24 years (r = 0.098, n = 735, *p* = 0.008), remaining below the corrected threshold after both Benjamini–Hochberg and Bonferroni correction (q = 0.032 and Bonferroni-adjusted *p* = 0.032). This relationship remained evident after adjustment for BMI at 8 years and after further adjustment for log-transformed exact age at the age-24 clinic visit. In these multivariable models, BMI at 8 years showed little evidence of association with INSL3 at 24 years, whereas log-transformed exact age at the age-24 clinic visit showed a negative association with INSL3. In contrast, no meaningful associations were observed for fatty-acid-related measures. Among amino acids and dietary trace-element measures, glutamine at 17 years showed a weak nominal positive association with INSL3 at 24 years, but this did not remain below the threshold after multiple-testing correction. **Conclusions:** Childhood vitamin D status, particularly at 9 years of age, may represent an early-life factor associated with variation in adult Leydig cell functional capacity. Fatty acid-related measures showed no clear associations, while glutamine yielded only a weak exploratory signal. These findings support the concept that the childhood metabolic environment may influence the developmental establishment of adult Leydig cell capacity.

## 1. Introduction

In men, testicular Leydig cells produce most of the testosterone required throughout adult life, and both their number and degree of differentiation are of critical importance for male reproductive and general health. Circulating insulin-like peptide 3 (INSL3) is particularly valuable for assessing Leydig cell functional capacity because it is produced exclusively by mature Leydig cells and secreted constitutively, making it less susceptible than testosterone to acute fluctuations [1]. As a result, INSL3 serves as a biomarker reflecting the size and differentiation status of the existing Leydig cell population [1]. Longitudinal INSL3 measurement confirms the notion that the adult Leydig cell population remains stable over many years [2], implying that the numbers of Leydig cells acquired in young adulthood more or less persist into old age with little, if any, attrition or replacement [3]. In adult men, circulating INSL3 levels vary more than 10-fold between individuals, and lower concentrations have been associated with hypogonadism and greater age-related morbidity [4]. Understanding the factors that establish this variation early in life is therefore important for clarifying how male health may be programmed across the lifespan.

The developmental timing of Leydig cell biology makes this question particularly important. In the human testis, after the relative quiescent period that follows fetal and early postnatal Leydig cell activity, the adult-type Leydig cell lineage begins to be re-established in late childhood and early puberty. Studies have shown that partially differentiated prepubertal Leydig cells can be detected from around 8–9 years of age, and that mature adult Leydig cells may begin to appear from approximately 8–10 years onwards [5,6]. In particular, it is difficult to distinguish whether the emergence of adult-type Leydig cells reflects proliferation of Leydig cell precursors, differentiation of existing immature cells, or both. Therefore, childhood and early adolescent exposures associated with later INSL3 should be interpreted as potential influences on the maturation and functional establishment of the adult Leydig cell compartment, rather than as direct evidence for altered Leydig cell proliferation. It is therefore biologically highly plausible that the metabolic and nutritional environment during childhood and early adolescence could influence the adult Leydig cell “set-point” that becomes established later in life.

Within this framework, analysing INSL3 provides an opportunity not only to assess transient hormonal variation, but also to indirectly interrogate how the adult Leydig cell population has been developmentally established. In other words, if an exposure occurring during childhood or adolescence is associated with later INSL3 levels, it is plausible that such an exposure may have influenced the number of adult Leydig cells, their degree of differentiation, or both. This perspective is important because it shifts the focus away from acutely regulated end-products such as testosterone and towards adult Leydig cell functional capacity itself [1].

This approach has been supported by our previous studies in the ALSPAC cohort. In our first study, we showed that INSL3 at 17 years had not yet fully stabilised, whereas by 24 years it reflected a more stable adult Leydig cell state, with peak INSL3 levels in this cohort being reached at approximately 22 years of age [3]. In the same study, we concluded that a substantial proportion of the variance in INSL3 at 24 years was already established by age 17, implying that the key determining influences must largely have arisen earlier. In a subsequent study, maternal variables showed only limited associations, whereas childhood/adolescent BMI and early childhood infections showed more meaningful relationships with young adult INSL3. Taken together, these findings strengthen the hypothesis that the factors determining adult Leydig cell capacity should be sought within the developmental environment of childhood and adolescence. Within this developmental framework, nutritional and metabolic markers such as vitamin D, fatty acid profile, leucine, glutamine, and zinc are of particular interest, since all of these variables are linked to fundamental biological processes that may determine adult Leydig cell functional capacity.

Within this developmental framework, vitamin D is a particularly plausible candidate, as the demonstration of the vitamin D receptor (VDR) and vitamin D-metabolising enzymes in the human testis suggests that this pathway has direct relevance for testicular biology [7,8]. Alongside vitamin D, fatty acids are also plausible candidates, as associations have been reported in young healthy men between fatty acid intake, reproductive hormone levels, and testicular volume, while experimental studies have shown that Leydig cells utilise cholesterol esters enriched in omega-6 highly unsaturated fatty acids to support steroid hormone production [9,10]. Amino acids provide a further potential nutritional link. Leucine is of particular interest because the mTOR pathway is one of the principal nutrient-sensitive regulators of cell growth, proliferation, and metabolism, and also plays an important role in the male reproductive system; leucine is one of the best-established nutritional activators of this pathway [11]. Consistent with this, dietary leucine supplementation has been reported in an experimental model to enhance testicular development and increase testicular mTOR/p-mTOR-related signalling [12]. Glutamine, in turn, is linked to cellular energy balance and redox defence, whereas zinc is closely related to steroidogenesis and androgen production; experimental data suggest that glutamine can support testicular glutathione-dependent antioxidant defence, while human studies indicate that lower serum zinc concentrations may be associated with lower testosterone levels [13,14].

Together, these nutritional and metabolic markers may reflect not only general nutritional status during childhood and adolescence, but also biological pathways capable of influencing the proliferation, differentiation, and steroidogenic capacity of the adult Leydig cell population as it becomes established during puberty. Relating vitamin D, fatty acids, amino acids, and dietary trace-element intake to INSL3 therefore provides a biologically rational approach to investigating early-life correlates of adult Leydig cell functional capacity.

## 2. Materials and Methods

The Avon Longitudinal Study of Parents and Children (ALSPAC) is an ongoing population-based birth cohort established in the former county of Avon, UK, to investigate factors influencing health and development across the life course [15]. Pregnant women resident in Avon with expected delivery dates between 1 April 1991 and 31 December 1992 were eligible for enrolment. In total, 20,248 eligible pregnancies have been identified, of which 14,541 were enrolled in the initial cohort [15,16,17]. Because additional eligible participants were recruited when the offspring were approximately 7 years old, analyses using data collected from age 7 onwards are based on a larger sample of 15,447 pregnancies, resulting in 14,901 children alive at 1 year of age [17,18]. ALSPAC has generated a wide range of questionnaire, clinic, biological, dietary, and metabolic data. Please note that the study website contains details of all the data that are available through a fully searchable data dictionary and variable search tool. For the present analysis, we used data from 1781 young male participants with relevant measurements available at different developmental time points. The wider enrolled ALSPAC offspring cohort is predominantly White, with 96.1% of participants with linked ethnicity information classified as White [15]; however, ethnicity information was not available with sufficient completeness and subgroup representation in the present analytical dataset to support reliable ethnicity-specific analyses. Study data were collected and managed using REDCap electronic data capture tools hosted at the University of Bristol. REDCap is a secure, web-based software platform designed to support data capture for research studies [19].

At the 17- and 24-year clinic assessments, peripheral venous blood samples were obtained and processed to generate serum or plasma aliquots, which were stored frozen at −80 °C until analysis. INSL3 concentrations were subsequently quantified at both time points using a well-validated time-resolved fluorescent immunoassay. The analytical limit of detection was 20 pg/mL, with intra- and inter-plate coefficients of variation of less than 9%. Childhood BMI at 8 years was obtained from mother-reported questionnaire data. In ALSPAC, questionnaires were sent to mothers annually when participants were between 8 and 16 years of age and included anthropometric information from which BMI variables were derived. BMI at 8 years was selected as the childhood adiposity measure closest to the age-9 vitamin D assessment.

### 2.1. Vitamin D Measurements

Serum concentrations of 25-hydroxyvitamin D2 [25(OH)D2] and 25-hydroxyvitamin D3 [25(OH)D3] were measured at ages 7, 9, and 11 years. Following protein precipitation, the analytes and a deuterated internal standard were extracted from serum using Isolute C18 solid-phase extraction cartridges. Potentially interfering compounds were removed by initial elution with 50% methanol, after which vitamin D metabolites were eluted with acetonitrile containing 10% tetrahydrofuran. Dried extracts were reconstituted and analysed by HPLC-MS/MS operating in multiple reaction monitoring (MRM) mode. The MRM transitions (m/z) used for 25(OH)D2, 25(OH)D3, and hexa-deuterated 25(OH)D3 were 413.2→395.3, 401.1→383.3, and 407.5→107.2, respectively. The assay working range was 2.5–624 nmol/L for both analytes, with coefficients of variation below 10% across the full range. Total 25(OH)D was calculated as the sum of 25(OH)D2 and 25(OH)D3.

### 2.2. Fatty Acid and Amino Acid Measurements

Fatty acid-related traits and low-molecular-weight metabolites were measured in plasma samples using high-throughput proton nuclear magnetic resonance (^1H-NMR) spectroscopy. These analyses were carried out in collaboration with Professor Mika Ala-Korpela and his laboratory in Finland. ^1H-NMR data were acquired across three molecular windows: lipoprotein lipids (LIPO), low-molecular-weight metabolites (LMWMs), and lipid extracts (LIPID). The LIPO and LMWM windows were measured in native plasma at 500 MHz, whereas the LIPID window was obtained from lipid-extracted plasma samples at 600 MHz. The LIPO window was acquired using a Bruker noesypresat pulse sequence with an automatically calibrated 90° pulse, a mixing time of 10 ms, and an irradiation field of 25 Hz for water suppression. The LMWM window was acquired using a water-suppressed Bruker CPMG pulse sequence, while the LIPID window was recorded using a standard one-dimensional (1D) sequence. Fatty acid-related measures were derived from the lipid and lipoprotein windows, whereas amino acids and other small metabolites were quantified from the LMWM window [20]. The available NMR-derived fatty-acid variables represented broad circulating fatty-acid classes and did not distinguish omega-3 from omega-6 fatty acids or provide individual fatty-acid measures for the present analyses.

### 2.3. Assessment of Maternal and Childhood Selenium and Zinc Intake

In this study, dietary information was obtained both from maternal questionnaires during pregnancy and from childhood dietary records. Maternal selenium intake was estimated from a food frequency questionnaire completed by mothers at approximately 32 weeks of gestation, which assessed the habitual consumption of a wide range of foods and drinks. This questionnaire had previously been shown to provide reasonable estimates of mean maternal nutrient intake, with values comparable to those reported for women in the British National Diet and Nutrition Survey for adults [21].

Childhood zinc intake was assessed at ages 7, 10, and 13.5 years, whereas childhood selenium intake was assessed at ages 7 and 10 years. At each time point, dietary intake was evaluated using three 1-day food diaries completed by carers a few days before the relevant clinic visit. Carers were asked to record all food and drink consumed by the child on two weekdays and one weekend day; these days were not required to be consecutive. The food diaries were coded by a trained nutritionist using the DIDO (Diet In, Data Out) program, and dietary selenium and zinc intakes were estimated from these records.

### 2.4. Statistical Analysis

All statistical analyses were performed using R (version 2024.05.0) and GraphPad Prism (version 10.6.1). Variables with evident positive skew were log-transformed before analysis to improve distributional normality. Initial screening of associations between candidate nutritional/metabolic variables and circulating INSL3 at 17 and 24 years was carried out using bivariate correlation analysis. Variables showing signals of interest were then examined further using multiple linear regression models.

To account for multiple testing in the bivariate screening analyses, the Benjamini–Hochberg procedure was used to control the false discovery rate at 5%. Corrections were applied separately for INSL3 outcomes at 17 and 24 years and within biologically defined exposure domains: vitamin D measures (four tests per outcome), fatty-acid-related measures (12 tests per outcome), and amino-acid and dietary trace-element measures (14 tests per outcome). These domains were considered separately because they represented distinct biological hypotheses and measurement groups. For the vitamin D domain, Bonferroni-adjusted *p* values were additionally examined as a sensitivity analysis.

For the main vitamin D analysis, log-transformed INSL3 at 24 years was used as the outcome variable, with log-transformed vitamin D at 9 years used as the primary predictor. Although this assessment is referred to as the age-24 clinic, participants did not all attend at exactly 24 years of age, and the actual age at attendance could vary by approximately one year in either direction. Therefore, exact age at the age-24 clinic visit, recorded in months, was included as a covariate in the relevant regression models to account for variation in the timing of INSL3 measurement. The first multivariable model adjusted for log-transformed BMI at 8 years, selected as the childhood adiposity measure closest to the age-9 vitamin D assessment. A second model additionally adjusted for log-transformed exact age at the age-24 clinic visit, recorded in months, to account for variation in the timing of INSL3 measurement at the age-24 clinic assessment. For the glutamine analysis, log-transformed glutamine at 17 years was examined in relation to log-transformed INSL3 at 24 years after adjustment for log-transformed exact age at the age-24 clinic visit. Regression analyses were based on complete-case data for the variables included in each model. To evaluate whether the reduction in sample size influenced the vitamin D association, the unadjusted association between vitamin D at 9 years and INSL3 at 24 years was repeated among the same 409 participants included in the complete-case regression models. The assumptions of the linear regression models were assessed by visual inspection of Q–Q plots of residuals and residuals-versus-fitted-value plots. These assessments indicated no material departures from residual normality or homoscedasticity. Independence of observations was supported by each participant contributing only one observation to each regression model, and variance inflation factors indicated no concerning multicollinearity among the independent variables. All statistical tests were two-sided. Effect estimates, 95% confidence intervals, and *p*-values were considered together to assess the strength and precision of evidence for associations. Given the exploratory screening of multiple nutritional and metabolic variables, findings were interpreted cautiously, with emphasis on effect size, consistency across developmental time points, and biological plausibility rather than on a fixed *p*-value threshold.

Participants were included in each bivariate analysis when data were available for both the relevant exposure and the corresponding INSL3 outcome. Therefore, the analytical sample was defined separately for each exposure–outcome pair, and the resulting sample sizes are reported in the relevant tables. Regression models included participants with complete data for the relevant exposure, INSL3 outcome, and all covariates included in the model. Missing values were not imputed because the variables were collected at different developmental time points and no prespecified imputation model was available.

### 2.5. Ethical Considerations

Ethical approval for the study was obtained from the ALSPAC Ethics and Law Committee and the relevant Local Research Ethics Committees. Consent for the collection and use of biological samples was obtained from all participants in accordance with the Human Tissue Act 2004. The use of questionnaire-derived and clinic-derived data was based on informed consent obtained from participants in line with the ALSPAC Ethics and Law Committee guidance applicable at the time of data collection. All data used in the present study were fully anonymised (http://www.bristol.ac.uk/alspac/researchers/research-ethics/) (accessed on 4 June 2026). Further details of the cohort, study design, and available variables have been published previously and are available through the ALSPAC website, including its searchable data dictionary and variable search facility (http://www.bristol.ac.uk/alspac/researchers/our-data/) (accessed on 4 June 2026).

## 3. Results

### 3.1. Fatty Acids

Bivariate correlation analysis provided little evidence of association between circulating INSL3 concentrations at either 17 or 24 years and fatty acid-related parameters measured at 7, 8.5, 15.5, or 17 years. This pattern was consistent across polyunsaturated fatty acids (PUFAs), monounsaturated fatty acids (MUFAs), and saturated fatty acids (SFAs), with correlation coefficients remaining close to zero throughout. At age 17 years, all fatty acid-related correlations with INSL3 were weak and provided little evidence of association, including PUFA, MUFA, and SFA at all time points examined in Table 1. Similarly, little evidence of association was observed between these fatty acid parameters and INSL3 at age 24 years. Overall, these findings do not support a meaningful relationship between childhood or adolescent fatty acid profiles and later circulating INSL3 concentrations.

### 3.2. Vitamin D

As shown in Table 2, vitamin D at 9 years was positively correlated with INSL3 at 24 years (r = 0.098, n = 735, unadjusted *p* = 0.008). This association remained below the corrected threshold after Benjamini–Hochberg correction (q = 0.032) and in the Bonferroni sensitivity analysis (adjusted *p* = 0.032). Vitamin D measured during pregnancy, at 7 years, and at 11 years showed no notable associations with INSL3 at 24 years. Similarly, none of the vitamin D measures showed an association with INSL3 at 17 years. Therefore, vitamin D at 9 years was taken forward for further multivariable analysis in relation to INSL3 at 24 years.

When the unadjusted analysis was restricted to the same 409 participants included in the complete-case regression models, the association between vitamin D at 9 years and INSL3 at 24 years remained evident (r = 0.124, 95% CI 0.027 to 0.218, *p* = 0.012; R^2^ = 0.0154). The estimate was comparable to the association observed in the larger bivariate sample (r = 0.098, n = 735), indicating that the association was not generated solely by restriction to the complete-case sample. Nevertheless, possible selection bias resulting from missing covariate data cannot be excluded. Table 3 shows that log-transformed vitamin D at 9 years remained positively associated with log-transformed INSL3 measured at the age-24 clinic assessment after adjustment for log-transformed BMI at 8 years (β = 0.213, 95% CI 0.048 to 0.379, *p* = 0.012). Thus, the association between vitamin D at 9 years and INSL3 at the age-24 clinic assessment was not explained by childhood BMI measured close to the vitamin D assessment.

When log-transformed exact age at the age-24 clinic visit was added to the model including log-transformed vitamin D at 9 years and log-transformed BMI at 8 years, vitamin D at 9 years remained positively associated with log-transformed INSL3 measured at the age-24 clinic assessment (β = 0.176, 95% CI 0.011 to 0.341, *p* = 0.037). BMI at 8 years was not associated with INSL3 in this model (β = 0.013, 95% CI −0.306 to 0.333, *p* = 0.936), whereas exact age at the age-24 clinic visit showed a marked negative association with INSL3 (β = −2.893, 95% CI −4.606 to −1.181, *p* = 0.001). Taken together, these findings indicate that the association between vitamin D at 9 years and INSL3 at the age-24 clinic assessment persisted after adjustment for childhood BMI and variation in the timing of outcome assessment (Table 4).

**Figure 1:**
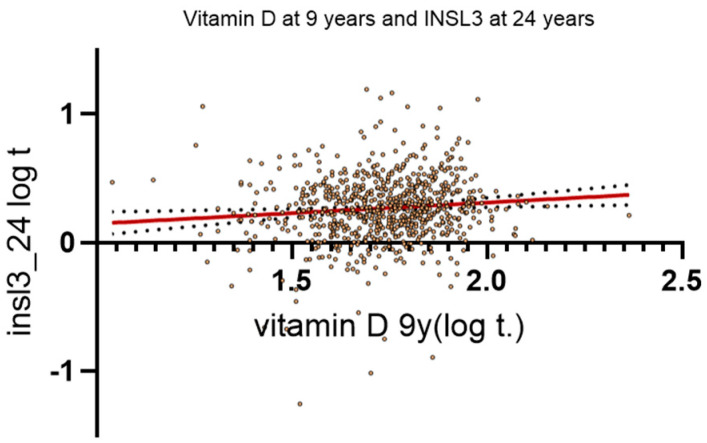
Association between log-transformed vitamin D at 9 years and log-transformed INSL3 at 24 years.

The relationship between log-transformed vitamin D at 9 years and log-transformed INSL3 at 24 years is shown in Figure 1.

### 3.3. Amino Acids and Dietary Trace-Element Intake

As shown in Table 5, bivariate correlation analysis revealed no noteworthy associations between INSL3 at 17 years and any of the amino acid or dietary trace-element intake measures examined. In particular, glutamine measured at 7, 8.5, 15.5, or 17 years, leucine measured at the same ages, dietary zinc intake at 7, 10, and 13.5 years, and dietary selenium intake during pregnancy, at 7 years, and at 10 years were all unrelated to INSL3 concentrations at 17 years. At 24 years, most of these parameters likewise showed no association with INSL3. Glutamine at 17 years showed a weak nominal positive correlation with INSL3 at 24 years (r = 0.080, n = 741, unadjusted *p* = 0.030); however, this association did not remain below the corrected threshold after Benjamini–Hochberg correction (q = 0.417) and was therefore interpreted as exploratory. There was little evidence of association for leucine, dietary zinc intake, or dietary selenium intake at any time point. Overall, Table 5 indicates that amino acids and dietary trace-element intake showed little evidence of a consistent relationship with later circulating INSL3 concentrations, apart from a modest association for glutamine at 17 years.

Because age at the 24-year clinic visit had previously been shown to contribute to variation in INSL3 concentration, the association between glutamine at 17 years and INSL3 at 24 years was further examined in an age-adjusted multiple regression model. As shown in Table 6, log-transformed glutamine at 17 years remained positively associated with INSL3 at 24 years after adjustment for log-transformed age at the age-24 clinic visit (β = 0.4114, 95% CI 0.0035 to 0.8192, *p* = 0.0481). Log-transformed age at clinic visit again showed a negative association with INSL3 at 24 years (β = −2.680, 95% CI −4.011 to −1.350, *p* < 0.0001). Thus, Table 6 indicates a weak nominal association after age adjustment; however, because the underlying correlation did not remain below the corrected threshold after multiple-testing correction, this finding should be regarded as exploratory rather than as independent evidence of a glutamine effect.

## 4. Discussion

The principal finding of the present study is that, among the nutritional and metabolic markers measured during childhood and adolescence, vitamin D at 9 years showed the most consistent positive association with INSL3 at 24 years, a biomarker of young adult Leydig cell functional capacity [1]. This relationship emerged in bivariate analyses and remained evident in multivariable log-transformed models after adjustment for BMI at 8 years and log-transformed exact age at the age-24 clinic visit. In these models, BMI at 8 years was not associated with INSL3 at the age-24 clinic assessment, whereas log-transformed exact age at clinic visit was negatively associated with INSL3. In contrast, fatty acid-related parameters showed no evidence of association, while among amino acids and dietary trace-element measures only glutamine at 17 years yielded a weak and borderline age-adjusted signal. Taken together, these findings suggest that childhood vitamin D status deserves particular consideration as a potential early-life correlate of adult Leydig cell functional capacity.

This conclusion is conceptually consistent with our previous work in the ALSPAC cohort. In our first study, we showed that INSL3 at 17 years had not yet fully stabilised, whereas by 24 years it reflected a more stable adult Leydig cell state, with peak INSL3 in this cohort being reached at approximately 22 years of age [3]. We also showed that nevertheless a substantial component of the variance in INSL3 at 24 years was already established by age 17, implying that the determining influences must largely have acted earlier in development. In a subsequent study, maternal variables proved to be relatively uninformative, whereas childhood/adolescent BMI and early childhood infections showed more meaningful associations with young adult INSL3. The present study extends that developmental framework by asking whether childhood nutritional and metabolic markers may represent another class of early-life influences on the establishment of adult Leydig cell capacity. Within that framework, vitamin D emerged as the most statistically consistent signal, although its effect size and explained variance were small.

The importance of the vitamin D finding lies not only in the statistical evidence supporting the association, but also in its biological coherence. Because INSL3 is secreted constitutively by mature Leydig cells, variation in this biomarker is likely to reflect the total functional capacity of the Leydig cell population rather than short-term endocrine fluctuation [1]. A childhood exposure associated with later INSL3 may therefore point towards an influence on the developmental programming of the adult Leydig cell population itself, rather than on transient regulation of testosterone secretion. In this context, vitamin D is biologically plausible. The presence of the vitamin D receptor and vitamin D-metabolising enzymes in the human testis suggests direct testicular relevance, and previous human and ex vivo observations have linked vitamin D deficiency with reduced Leydig responsiveness and active vitamin D with stimulation of testosterone production [7]. This potential link is further supported by evidence that Leydig cells express CYP2R1, a key vitamin D 25-hydroxylase, and that gonadotrophin signalling through LH/hCG may modulate this pathway; however, acute hCG stimulation does not appear to increase circulating 25(OH)D, suggesting that any gonadotrophin-related influence on vitamin D metabolism is more likely to reflect chronic or developmental Leydig cell function rather than an acute endocrine response [22]. Thus, it is reasonable to propose that adequate vitamin D status during late childhood could influence the proliferative, differentiative, or steroidogenic maturation of the Leydig cell lineage during the pubertal transition.

Previous work in the ALSPAC cohort indicates that childhood vitamin D status should not be interpreted solely as a marker of dietary vitamin D intake. Serum 25-hydroxyvitamin D [25(OH)D] reflects both cutaneous synthesis following UVB exposure and dietary or supplemental sources. In ALSPAC with 25(OH)D3 and 25(OH)D2 measured at a mean age of 9.9 years, Tolppanen et al. reported that vitamin D deficiency was associated with winter season, less time spent outdoors, lower socioeconomic position, non-white ethnicity, older age, more advanced pubertal stage, and female sex [23]. The same study also identified BMI measured at the time of blood sampling, physical activity, household income, and maternal education as factors associated with 25(OH)D3 and/or 25(OH)D2 concentrations. Importantly, this supports the use of BMI at 8 years in the present analysis as the closest available childhood adiposity measure to the age-9 vitamin D assessment. Therefore, childhood 25(OH)D concentrations are likely to represent an integrated marker of UVB exposure, adiposity, pubertal maturation, socioeconomic conditions, and lifestyle, rather than dietary intake alone.

At the same time, the present findings require careful interpretation. The observed effect size is modest, which argues against vitamin D being viewed as a dominant or isolated determinant of adult Leydig cell capacity. Compared with the previously observed associations between childhood/adolescent BMI and young adult INSL3, the vitamin D association appears modest, both in terms of effect size and explained variance. It should therefore be interpreted not as a dominant determinant of adult Leydig cell functional capacity, but as an additional early-life correlate within the same broader developmental framework. Moreover, the clearest signal was observed for vitamin D measured at 9 years. Although the present data do not establish age 9 as a uniquely sensitive window, this measurement coincides with a biologically relevant developmental period: adult-type Leydig cell precursors are thought to begin proliferating and differentiating at approximately 8–9 years, before the progressive appearance of mature steroidogenic characteristics during the subsequent pubertal years [5,6]. Together with the presence of VDR and vitamin D-metabolising machinery in human Leydig cells and testicular tissue [7,8], this provides a biologically plausible basis for proposing that vitamin D status during this period could influence early Leydig cell differentiation or steroidogenic maturation. This interpretation should nevertheless be regarded as a developmental hypothesis rather than proof of an age-specific effect.

The multivariable models further support the relevance of this association. The persistence of the vitamin D signal after adjustment for BMI at 8 years suggests that the relationship is not explained simply by childhood BMI measured close to the vitamin D assessment. Its persistence after additional adjustment for log-transformed exact age at the age-24 clinic visit is also important, because our previous work showed that actual age at this clinic visit contributes to variation in INSL3 at 24 years and may reflect the early decline that follows the post-pubertal peak. The fact that vitamin D remained positively associated after accounting for both childhood BMI and this timing variable argues against the signal being merely an artefact of childhood adiposity or slight differences in the timing of outcome assessment.

By contrast, the results for fatty acids were largely null. No meaningful associations were observed between PUFA, MUFA, or SFA measures and INSL3 at either 17 or 24 years. Although this may appear somewhat disappointing in light of mechanistic work linking lipid handling to steroidogenesis, several explanations are possible [9,10]. Plasma fatty acid profiles or NMR-derived lipid traits may not adequately capture the lipid microenvironment that is most relevant within the testicular interstitium. In addition, any real effects of fatty acid composition on Leydig cell biology may be subtle, highly context-dependent, and difficult to detect in a population-based cohort using single time-point measures. Thus, the present negative findings do not necessarily imply that fatty acids are biologically irrelevant, but rather that they did not emerge here as major explanatory variables for variation in young adult INSL3.

The amino acid and dietary trace-element intake findings were also mostly negative. Among the variables examined, only glutamine at 17 years showed a weak positive association with INSL3 at 24 years, with limited evidence that this persisted after adjustment for log-transformed exact age at clinic visit. However, this signal must be interpreted cautiously. The effect size was small and the variance explained by the model was very limited, indicating that glutamine, even if real, is unlikely to be a major determinant. Moreover, corresponding associations were not observed at earlier ages, making the biological meaning of the signal uncertain. At present, the glutamine result is best viewed as exploratory and hypothesis-generating rather than as a robust parallel finding to the vitamin D result. Similarly, the absence of clear associations for leucine, dietary zinc intake, or dietary selenium intake does not rule out biological relevance, but suggests that within this cohort and with these measurements they did not emerge as strong predictors of later Leydig cell functional capacity.

This study has several strengths. First, the use of a large, deeply phenotyped longitudinal cohort allowed the investigation of multiple childhood and adolescent exposures in relation to a young adult biomarker of Leydig cell function. Second, the regression framework incorporated BMI at 8 years, a childhood adiposity measure close to the vitamin D assessment, and log-transformed exact age at the age-24 clinic visit, both of which are relevant to the interpretation of the vitamin D-INSL3 association. The potential biological and clinical implications of these findings should therefore be considered cautiously. Our data suggest that childhood vitamin D status may contribute to between-individual variation in young adult Leydig cell functional capacity, but they do not justify a direct clinical inference or intervention recommendation on their own. What they do is support the broader concept that the late childhood and early adolescent metabolic environment may influence the pubertal establishment of the adult Leydig cell population. Future work should investigate childhood vitamin D status alongside pubertal timing, gonadotrophin dynamics, testicular growth, and more direct steroidogenic outcomes. Similarly, exploratory signals such as that seen for glutamine should be tested in independent datasets and in more detailed metabolomic frameworks.

Several limitations should also be acknowledged. First, the observational design does not allow causal inference, and the observed associations should be interpreted as exploratory. Second, multiple nutritional and metabolic variables were examined, increasing the possibility of chance findings, particularly for weaker signals such as glutamine. Third, the available sample size varied across exposure time points, and regression models were based on complete cases. This may have reduced statistical power and introduced selection bias if data completeness was related to the exposures, outcomes, or participant characteristics; consequently, comparability across analyses may be limited. Fourth, residual confounding by factors such as season of blood sampling, socioeconomic position, physical activity, pubertal timing, and other lifestyle-related variables cannot be excluded. Finally, single time-point measurements of circulating metabolites or dietary intake may not fully capture long-term nutritional status.

## 5. Conclusions

Among the nutritional and metabolic variables examined here, vitamin D at 9 years showed the most consistent positive association with INSL3 at 24 years. This relationship remained evident after adjustment for BMI at 8 years and log-transformed exact age at the age-24 clinic visit. Fatty acid-related variables showed no meaningful associations, while glutamine yielded only a weak exploratory signal. These findings suggest that childhood vitamin D status may be associated with later variation in adult Leydig cell functional capacity, while also highlighting the need for further epidemiological replication and mechanistic validation.

## Figures and Tables

**Table 1:** Bivariate correlations between fatty-acid-related parameters and circulating INSL3 at 17 and 24 years.

	INSL3 at 17 Years			INSL3 at 24 Years		
	Pearson Correlation	*p* Value	N	Pearson Correlation	*p* Value	N
PUFA (7 y)	−0.015	0.663	857	0.035	0.337	775
PUFA (8.5 y)	0.001	0.991	153	0.015	0.873	118
PUFA (15.5 y)	0.027	0.440	830	−0.005	0.885	700
PUFA (17 y)	−0.013	0.630	1229	0.033	0.370	739
MUFA (7 y)	0.037	0.275	856	0.013	0.708	775
MUFA (8.5 y)	−0.027	0.739	153	0.037	0.695	118
MUFA (15.5 y)	0.059	0.091	830	0.005	0.889	701
MUFA (17 y)	0.037	0.195	1229	0.040	0.283	739
SFA (7 y)	−0.013	0.706	856	0.040	0.263	775
SFA (8.5 y)	0.019	0.812	153	0.031	0.736	118
SFA (15.5 y)	0.054	0.123	830	0.051	0.179	701
SFA (17 y)	−0.008	0.778	1229	0.031	0.397	739

Abbreviations: INSL3, insulin-like peptide 3; PUFA, polyunsaturated fatty acids; MUFA, monounsaturated fatty acids; SFA, saturated fatty acids; N, number of participants with non-missing paired data for each correlation; y, years.

**Table 2:** Bivariate correlations between vitamin D measures at different developmental time points and circulating INSL3 at 17 and 24 years.

	INSL3 at 17 Years			INSL3 at 24 Years		
	Pearson Correlation	*p* Value	N	Pearson Correlation	*p* Value	N
Vitamin D (dur. pregnancy)	−0.039	0.280	752	−0.054	0.160	686
Vitamin D 7 y	−0.016	0.893	63	0.043	0.690	90
Vitamin D 9 y	0.045	0.201	826	0.098	**0.008**	735
Vitamin D 11 y	0.025	0.763	147	0.130	0.147	127

Abbreviations: INSL3, insulin-like peptide 3; N, number of participants with non-missing paired data for each correlation; y, years. Significant correlations are highlighted in bold red font.

**Table 3:** Multiple linear regression model for log-transformed INSL3 at 24 years, adjusted for log-transformed BMI at 8 years.

Predictor	β Coefficient	95% CI	*p* Value
Intercept	−0.159	−0.655 to 0.337	0.529
Log(Vitamin D at 9 years)	0.213	0.048 to 0.379	**0.012**
Log(BMI at 8 y)	0.037	−0.286 to 0.360	0.821

Model N = 409; R^2^ = 0.01552; adjusted R^2^ = 0.01067. Significant regressions are highlighted in bold red font.

**Table 4:** Multiple linear regression model for log-transformed INSL3 at 24 years, adjusted for log-transformed BMI at 8 years and log-transformed exact age at the age-24 clinic visit.

Predictor	β Coefficient	95% CI	*p* Value
Intercept	7.079	2.767 to 11.39	**0.001**
Log(Vitamin D at 9 years)	0.176	0.011 to 0.341	**0.037**
Log(BMI at 8 y)	0.013	−0.306 to 0.333	0.936
Log(age at clinic visit, months)	−2.893	−4.606 to −1.181	**0.001**

Model N = 409; R^2^ = 0.04163; adjusted R^2^ = 0.03453. Significant regressions are highlighted in bold red font.

**Table 5:** Bivariate correlations between amino acids, dietary trace-element intake, and circulating INSL3 at 17 and 24 years.

	INSL3 at 17 Years			INSL3 at 24 Years		
Parameter	Pearson Correlation	*p* Value	N	Pearson Correlation	*p* Value	N
Glutamine (7 y)	−0.041	0.228	857	0.012	0.750	773
Glutamine (8.5 y)	−0.115	0.154	156	0.003	0.978	120
Glutamine (15.5 y)	−0.031	0.367	832	0.009	0.811	702
Glutamine (17 y)	−0.012	0.662	1233	0.080	**0.030**	741
Leucine (7 y)	0.007	0.828	858	0.001	0.997	774
Leucine (8.5 y)	0.031	0.699	156	−0.032	0.727	120
Leucine (15.5 y)	0.051	0.140	832	−0.009	0.813	703
Leucine (17 y)	0.010	0.736	1233	−0.035	0.344	741
Zinc intake (mg/day) (7 y)	−0.031	0.315	1029	−0.025	0.444	973
Zinc intake (mg/day) (10 y)	−0.033	0.261	1131	0.007	0.811	1053
Zinc intake (mg/day) (13.5 y)	−0.011	0.719	1076	−0.040	0.203	999
Selenium intake (µg/day) (dur. Preg.)	−0.019	0.503	1186	−0.006	0.850	1122
Selenium intake (µg/day) (7 y)	0.008	0.786	1029	−0.037	0.250	973
Selenium intake (µg/day) (10 y)	−0.041	0.168	1131	−0.013	0.680	1053

Abbreviations: INSL3, insulin-like peptide 3; N, number of participants with non-missing paired data for each correlation; y, years; dur. preg., during pregnancy. Significant correlations are highlighted in bold red font.

**Table 6:** Multiple linear regression model examining the association between log-transformed glutamine at 17 years and log-transformed INSL3 at 24 years after adjustment for log-transformed exact age at the age-24 clinic visit.

Predictor	β Coefficient	95% CI	*p* Value
Intercept	6.992	3.711 to 10.27	**<0.0001**
log(Glutamine at 17 years)	0.4114	0.0035 to 0.8192	**0.0481**
log(Age at clinic visit, months)	−2.680	−4.011 to −1.350	**<0.0001**

Model N = 740; R^2^ = 0.02708; adjusted R^2^ = 0.02444. Significant regressions are highlighted in bold red font.

## Data Availability

The data that support the findings of this study are available from the Avon Longitudinal Study of Parents and Children (ALSPAC), University of Bristol, subject to application and approval through the ALSPAC Executive Committee. The datasets are not publicly available due to ethical, legal, and participant confidentiality restrictions. Information on how to access ALSPAC data is available through the ALSPAC website, including its searchable data dictionary and variable search facility.

## Abbreviations

The following abbreviations are used in this manuscript:

ALSPAC	Avon Longitudinal Study of Parents and Children
BMI	Body mass index
CI	Confidence interva
DIDO	Diet In, Data Out
INSL3	Insulin-like peptide 3
LIPO	Lipoprotein lipids
LMWM	Low-molecular-weight metabolites
MRM	Multiple reaction monitoring
MUFA	Monounsaturated fatty acids
NMR	Nuclear magnetic resonance
PUFA	Polyunsaturated fatty acids
REDCap	Research Electronic Data Capture
SFA	Saturated fatty acids
VDR	Vitamin D receptor
